# Supplementary material for: Intrinsic excitation-inhibition imbalance affects medial prefrontal cortex differently in autistic men versus women
Source: eLife. 2020 Aug 4;9:e55684. doi: 10.7554/eLife.55684 (PMC7402681; doi:10.7554/eLife.55684)
Supplement: Supplementary file 1. [file elife-55684-supp1.docx]

| Parameter | Value |
| --- | --- |
| Population Firing Rate (E, I) | 2 Hz, 5 Hz |
| Population Size (E, I) | 8000, 2000 |
| Resting Membrane Potential | -65 mV |
| Reversal Potential (AMPA, GABA_A_) | 0 mV, -80 mV |
| Conductance Rise Time (AMPA, GABA_A_) | 0.1 ms, 0.5 ms |
| Conductance Decay Time (AMPA, GABA_A_) | 2 ms, 10 ms |
| E:I Ratio | 1:2 to 1:6 |

***Supplementary File 1: Parameters for E:I model:*** *Parameters utilized in the E:I model from Gao et al.,*^28^*, for simulating LFP data.*
